# Corrigendum: Chromatin structure and context-dependent sequence features control prime editing efficiency

**DOI:** 10.3389/fgene.2024.1391923

**Published:** 2024-03-11

**Authors:** Somang Kim, Jimmy B. Yuan, Wendy S. Woods, Destry A. Newton, Pablo Perez-Pinera, Jun S. Song

**Affiliations:** ^1^ Department of Physics, University of Illinois at Urbana-Champaign, Urbana, IL, United States; ^2^ Carl R. Woese Institute for Genomic Biology, University of Illinois at Urbana-Champaign, Urbana, IL, United States; ^3^ Department of Bioengineering, University of Illinois at Urbana-Champaign, Urbana, IL, United States; ^4^ Department of Biomedical and Translational Sciences, Carle-Illinois College of Medicine, University of Illinois at Urbana-Champaign, Urbana, IL, United States; ^5^ Cancer Center at Illinois, University of Illinois at Urbana-Champaign, Urbana, IL, United States; ^6^ Department of Molecular and Integrative Physiology, University of Illinois at Urbana-Champaign, Urbana, IL, United States; ^7^ Center for Theoretical Physics, Department of Physics, Massachusetts Institute of Technology, Cambridge, MA, United States; ^8^ Department of Statistics, Harvard University, Cambridge, MA, United States

**Keywords:** prime editing, CRISPR–Cas9, heterochromatin, nucleosome positioning, DNA-RNA hybridization, nucleotide preference, machine learning, neural network interpretation

In the published article, there was an error in **Supplementary Figure S3**. Even though the figure caption for Supplementary Figure S3 was correct, Figure S3 was inadvertently duplicated from Figure S2 and appeared as:


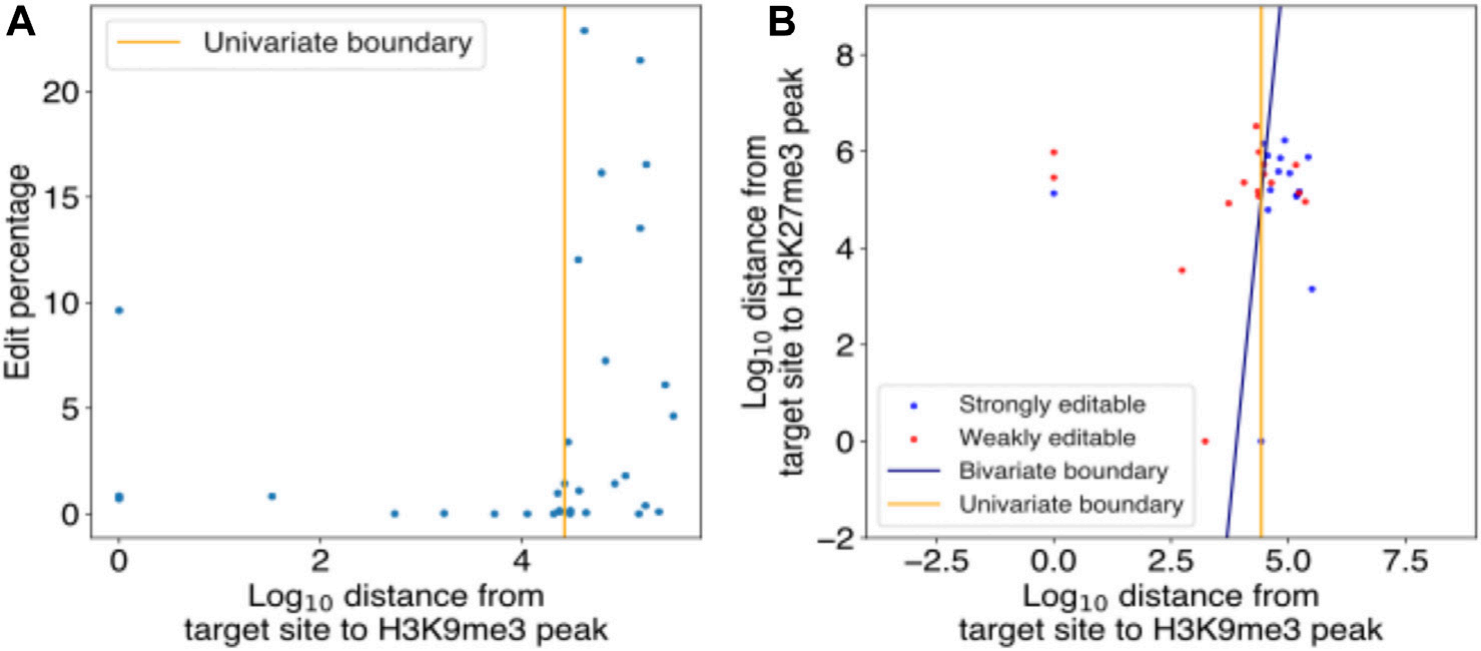


The correct figure appears below.


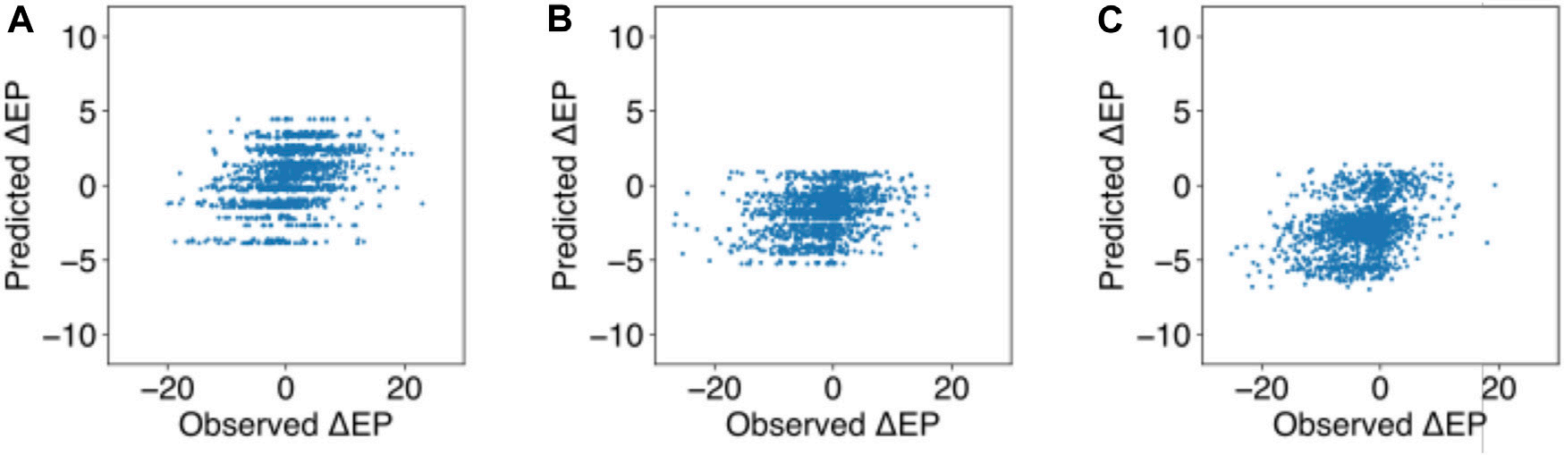


The authors apologize for this error and state that this does not change the scientific conclusions of the article in any way. The original article has been updated.

